# Expertise and processing distorted structure in chess

**DOI:** 10.3389/fnhum.2013.00825

**Published:** 2013-12-03

**Authors:** James C. Bartlett, Amy L. Boggan, Daniel C. Krawczyk

**Affiliations:** ^1^Program in Cognition and Neuroscience, School of Behavioral and Brain Sciences, The University of Texas at DallasRichardson, TX, USA; ^2^Department of Psychology, Young Harris College, Young HarrisGA, USA; ^3^Department of Psychiatry, The University of Texas Southwestern Medical CenterDallas, TX, USA

**Keywords:** chess, chunking, consciousness, expertise, meaning, prefrontal-parietal network, structure

## Abstract

A classic finding in research on human expertise and knowledge is that of enhanced memory for stimuli in a domain of expertise as compared to either stimuli outside that domain, or within-domain stimuli that have been degraded or distorted in some way. However, we do not understand how experts process degradation or distortion of stimuli within the expert domain (e.g., a face with the eyes, nose, and mouth in the wrong positions, or a chessboard with pieces placed randomly). Focusing on the domain of chess, we present new fMRI evidence that when experts view such distorted/within-domain stimuli, they engage an active search for structure—a kind of exploratory chunking—that involves a component of a prefrontal-parietal network linked to consciousness, attention and working memory.

## Introduction

A useful strategy for addressing a complex cognitive process is to present people with stimuli that engage that process, but which will also disrupt or interfere with it, creating errors or difficulties in its execution. A classic example is Bartlett's (1932) famous study of memory for an English translation of a North American (Inuit) folk tale called “The War of the Ghosts.” To his English participants, the story was strange with bizarre details and weird turns of events, and yet it was, quite clearly, a story. The well-known finding was that participants' reproductions of the story were distorted in a way that made them more coherent and plausible than the original story was, a phenomenon Bartlett called “rationalization” and which he attributed to “effort after meaning.” According to Bartlett (and many others since), rationalization and effort after meaning cannot be studied using meaningless stimuli, such as lists of nonsense syllables, because the relevant process—effort after meaning—will not be activated with such materials. At the same time, the cognitive effects of effort after meaning may be hard to discern with materials that are easy to interpret and readily assimilated with a person's prior knowledge. The effortful component of effort after meaning might be minimized in such cases.

Much more recently, Bor and colleagues (Bor and Owen, 2007; Bor, 2012; Bor and Seth, 2012) have proposed a conception of human consciousness that emphasizes the importance of frontal and parietal neural networks in the active search for patterns or chunks in stimulus displays, a process akin to Bartlett's concept of “effort after meaning.” A core observation comes from working memory tasks in which participants are able to improve their memory for a sequence of numbers by detecting that the sequence follows a rule or is a repetition of a previously studied sequence, allowing chunking on that basis. Chunking of such sequences is associated with extensive activation of a prefrontal-parietal network, as detected by fMRI. In considering these studies, it is important to keep in mind the distinction between active, strategic chunking and the identification of overlearned stimulus patterns such as familiar words and acronyms (e.g., dog, FBI, see Gobet et al., 2001, for an elaborated theoretical discussion). In Bor's theory, it is only active, strategic chunking that engages the prefrontal-parietal network. Thus, as chunking of stimuli in a given domain becomes automatized through practice—as might be the case in domains of expertise—the role of the prefrontal-parietal network will be decreased. For the sake of clarity, we will refer to such automatized chunking as “pattern recognition.”

The present paper is focused on stimuli that differ rather drastically from sequences of numbers, but which are well suited for the study of chunking and pattern recognition as a function of expertise. Specifically, we examine working memory for chessboard displays by master-level chess players, as well as, for comparison, less skilled players and novices at chess. Our findings suggest that chess masters engage at least one component of the prefrontal-parietal network in the service of chunking chessboard displays. However, they do this more with “scrambled” displays—boards on which the pieces are placed randomly—as opposed to normal displays that represent possible chess game positions. We argue that our findings are in line with the view that the prefrontal-parietal network is involved with strategic, non-automatized chunking, as opposed to the automatized pattern recognition that occurs with chess experts viewing normal chessboard displays.

The basis for our argument is perhaps the best known finding from over 60 years of research on expertise: Chess experts are much better at recalling normal displays than randomized displays and, with the former, their recall is much greater than that of novices or less skilled players (Chase and Simon, 1973). The result has been attributed to knowledge structures in long-term memory that allow experts to encode a normal chessboard as a relatively small number of patterns or groups, each including several pieces and their relative positions on the board. Novices lack such knowledge structures, and therefore, are unable to perform this type of grouping. Less skilled players have fewer such structures, and/or less elaborated structures, as compared to more skilled players. Therefore, less skilled players encode chessboards less effectively than more skilled players do, encoding fewer and smaller patterns (see, e.g., Gobet and Simon, 1996a,b).

The pattern recognition account of normal/random chessboard recall is virtually unchallenged in the expertise literature, and we accept it for purposes of the present research. However, little is known about what occurs when expert players encounter random chess displays. Gobet and Simon (1996a) marshaled evidence that recall of random chessboards is positively correlated with chess expertise, albeit more weakly than the recall of normal chessboards. This finding suggests that experts perform some degree of pattern recognition, even with random boards. Indeed, this finding (and others) was predicted by a computer model that was trained in identifying patterns of pieces in positions from master-level chess games (Gobet and Simon, 1996b). As the degree of simulated training increased, the model recognized more patterns in random boards.

Yet the processes that differentiate more and less skilled players when encoding random boards are not fully understood. The computer simulations of Gobet and Simon (1996b) give strong support to one hypothesis: Because chess experts have a huge data base of chess-piece configurations in long-term memory, they are more likely than less skilled players to recognize meaningful patterns that occur by chance in random games. However, Gobet and Simon noted that some of the chunks encoded by chess players are not meaningful in chess, citing the example of an expert noticing that three white pawns formed a diagonal from a1. Based on such observations, they concluded that “.. chessplayers may use special strategies to recall pieces on a board that is almost bare of familiar patterns” (p. 501). Similarly, Gobet and Simon (1996a) suggested that stronger and weaker players might differ in “the possession of strategies for coping with uncommon positions” (p. 161).

The present study addressed an idea that links the special-strategies hypothesis to the prefrontal-parietal network as conceptualized by Bor and Owen (2007). We propose that experts' processing of random chess displays differs from their processing of normal displays not only quantitatively (involving fewer and/or smaller patterns or groups), but qualitatively as well, engaging the prefrontal-parietal network in an active search for chunks. The chunks in question may include patterns of pieces identical or similar to what might occur in real chess games, as well illegal, strange, or highly unlikely patterns that are, nonetheless, encodable based on knowledge of chess (e.g., three white pawns on a diagonal from a1). The key claim is that experts engage this active, knowledge-based search process more than less skilled players do.

Our thinking departed from two recent studies comparing neural processing of chessboard displays—as well as faces and other stimuli—by experts and novices at chess (Bilalić et al., 2011; Krawczyk et al., 2011). Both studies were focused on the fusiform face area (FFA) in the ventral temporal cortex, due to its importance in the processing of faces. Further, both addressed the question of whether the FFA is better characterized as being face-specific—responding more to faces than non-faces—or expertise specific—responding to faces as well as other objects with which observers have high expertise. Using a standard working memory task (one-back), Bilalić et al. (2011, Experiment 1) reported that FFA activity was substantially greater for faces than chessboards, whether shown in standard upright orientation or upside-down. However, they also found that FFA activity in response to chessboards was greater among expert players than among novices at chess. Using a similar one-back working memory task, Krawczyk et al. (2011) also observed substantially higher FFA activation for faces than chessboards, though they found no evidence that FFA activation in response to chessboards was greater among experts than novices. Despite this discrepancy, the two studies converged in another respect: Both showed that FFA activation was as strong if not stronger with random chessboards than normal boards. In fact, in five of six conditions across Experiments 2 and 3 of the Bilalić et al. study, there was a statistically reliable interactive pattern such that experts showed stronger FFA activation with random boards than normal boards, while novices showed no difference. The normal-random comparison in the Krawczyk et al. study produced only non-significant trends in FFA activation, possibly due to the limited sample size.

The experiment reported here is the same as that reported by Krawczyk et al. (2011), with the addition of: (a) five new master-level chess experts, bringing the total expert sample to an n of 11, and (b) five midlevel players. According to their international Elo ratings (Elo, 1986), our master-level experts (*M* = 2469) had greater expertise than the Bilalić et al. experts (*M* = 2117). Our midlevel players had lower expertise, yet they were active players with national Elo ratings (*M* = 1501), and were substantially more skilled than our novice participants (*n* = 6), all of whom had played chess but did not do so regularly. Our goal was to further assess the strength of our prior observations, and, more importantly, to determine if experts' processing random boards produces not only an FFA response, but also activation of the prefrontal-parietal network previously described by Bor and Owen (2007). Our guiding hypothesis was that players with greater expertise would engage an active chunking strategy with the random chessboards to maximize their performance on the working memory task, and that their use of this strategy would involve activation in the prefrontal-parietal network, possibly extending to FFA regions due to top-down control effects (Corbetta and Shulman, 2002). Others have reported that prefrontal-parietal activation associated with working memory typically decreases in novices with practice up to a certain point at which functional reorganization occurs with greater expertise (Guida et al., 2012). Once an expert has achieved functional reorganization, he or she is more likely to access long term memory representations in the domain of expertise. Our expert players would likely fit this profile, showing *reduced* prefrontal-parietal activation during working memory for normal chess displays. Notwithstanding, an active chunking hypothesis predicts they will show *increased* prefrontal-parietal activation during working memory for random displays.

We planned to test our hypothesis with a whole-brain analysis as well as with more focused region-of-interest (ROI) comparisons based on coordinates for seven prefrontal-parietal regions provided by Bor and Owen (2007). These regions consisted of the anterior cingulate cortex (ACC) (Duncan and Owen, 2000), the left and right inferior parietal sulcus (IPS) (Duncan, 2006), the left and right ventrolateral prefrontal cortex (VLPFC) (Bor and Owen, 2007), and the left and right dorsolateral prefrontal cortex (DLPFC) (Bor et al., 2003, 2004). We also collected subjective reports of participants' chunking activity at the end of the experimental session. One goal was to determine whether any increment in activation for random boards as compared to normal boards extends throughout the entire prefrontal-parietal network, or is restricted to one or two components. A second goal was to test our assumption that such activation increments reflect an active search for chunks. To the extent that they do, these activation increments should show correlations with subjective reports of chunking.

In addition to the prefrontal-parietal ROI analyses, we also conducted ROI analyses for the left and right FFA. The goal here was to determine whether activation increments for random boards in the FFA (Bilalić et al., 2011) are linked to activation increments in the prefrontal-parietal network.

One final ROI analysis was aimed at linking our active-chunking hypothesis with accumulating evidence that highly expert individuals show activation in medial-temporal brain regions in working memory tasks with objects of expertise (see Campitelli et al., 2007 and Guida et al., 2012). The dominant explanation for these medial-temporal activations is that well-formed stimuli in a domain of expertise contain many familiar patterns that activate representations in long-term memory, allowing long-term memory to support performance in working memory tasks. Because normal chessboards contain more familiar patterns than randomized boards, expertise-related medial-temporal activations should be largely restricted to normal chessboards. Hence, while expertise-related prefrontal-parietal activations should be stronger with random boards than normal boards, expertise-related medial-temporal activations might be stronger with normal boards than random boards. We chose four medial-temporal ROIs—the left and right hippocampus and the left and right parahippocampal gyrus—to asses this possibility.

## Materials and methods

### Subjects

Subjects were 22 healthy, right-handed, male volunteers. Eleven subjects were chess experts recruited from the UT Dallas Chess Program, age 19–28 (*M* = 23 years). These subjects ranked within the top one percent of active tournament players (three Grandmasters; eight International Masters) at the time of the experiment. Subject expertise was substantiated by their competitive ratings (Elo range 2353–2570; *M* = 2469), their years playing chess (*M* = 16 years), and their tournament activity (*M* = 13 per year). Six of the remaining subjects were healthy males who were chess novices age 21–27 (*M* = 25 years). These subjects reported that they rarely played chess and had never participated in chess tournaments. Lastly, we included five players (age range 19–40, *M* = 24) who had some tournament experience in chess and were competitively rated (Elo range = 1332–1634; *M* = 1495), but did not approach the skill-level of the experts. Given the strong differences in expertise level between the experts and the other two groups (novices and individuals with some experience), we collapsed the non-expert groups forming a larger group of 11 individuals termed “less experienced players.” Notably there was no significant difference in behavioral accuracy on the chessboard conditions between the true novices and the individuals with some chess experience. This experimental protocol received approval from the Institutional Review Boards of The University of Texas at Dallas and UT Southwestern Medical Center. All subjects provided informed consent in accordance with the 1964 Declaration of Helsinki.

### MRI perception task

**Figure 1:**
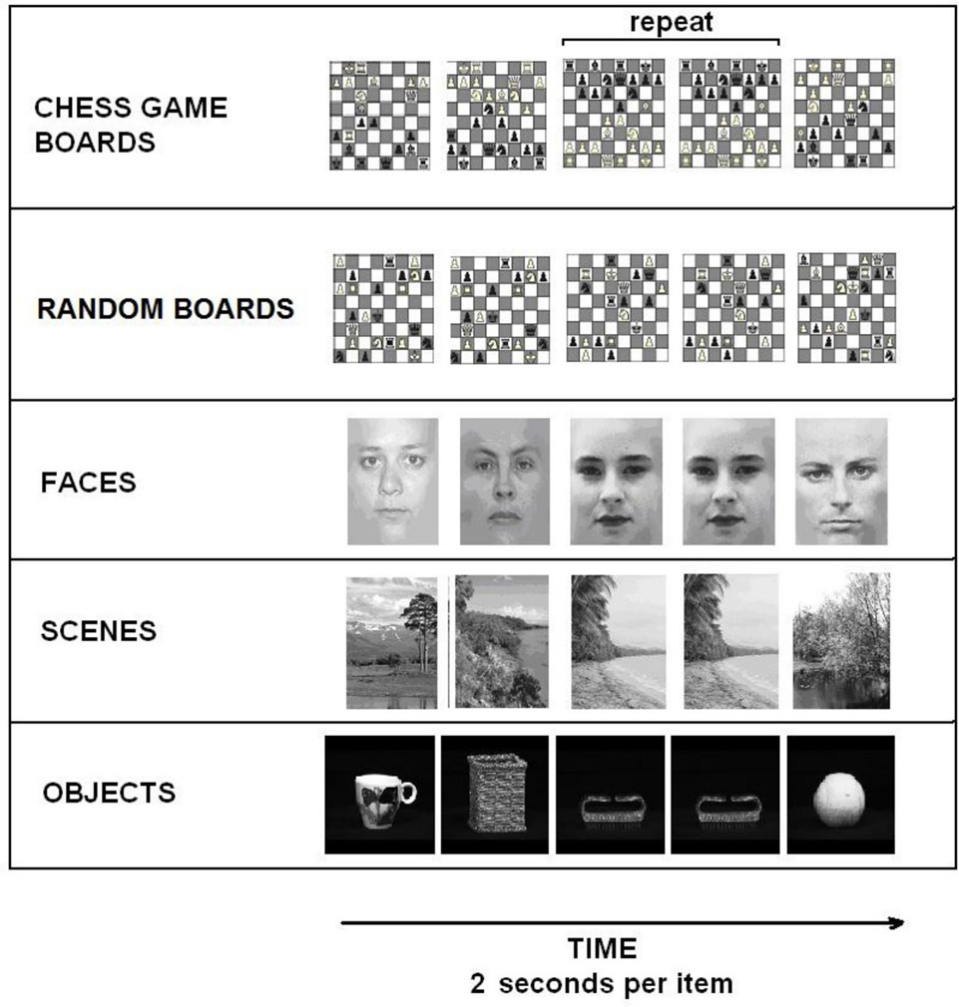
**Task figure showing each of the different condition blocks**. Images repeated twice in each stimulus block.

We used a one-back task previously described by Krawczyk et al. (2011). In the task we presented blocks of visual items and subjects judged whether each item was a repeated image or new image. Stimuli consisted of sets of images of chess boards from normal games, randomly positioned chess boards that could not occur in normal games, faces, everyday objects (from Geusebroek et al., 2005), and outdoor scenes (Figure 1). Images were presented in five runs of 8 blocks with 12 images per block, 2 s per image, 500 ms inter-stimulus-interval (ISI). Stimulus exposure times and ISIs were set to ensure that novices could reasonably perform the task given the degree of visual complexity present in chess board stimuli. Images were presented offset from center to the right or left in an alternating sequence to avoid apparent motion effects that occur in the chess conditions between non-matching stimuli that occur in sequence. One or two images repeated per block, and subjects were instructed to press both buttons (one in each hand) when a repeat was detected. Each block contained one type of image (e.g., faces) or was a fixation block lasting 30 s. Block order was presented in a pseudo-randomized manner.

### Post-session questionnaire

After the imaging task participants completed a questionnaire on which they rated the difficulty they experienced normal chessboards, random chessboards, faces, scenes, and objects on a 1 to 7 scale. They also estimated the average number of groupings they perceived with normal and random boards, the average number of chess pieces per grouping, and the average total number of pieces they tracked.

### Functional MRI acquisition and analysis

Images were acquired using a 3T Philips Achieva MRI scanner running a gradient echoplanar sequence (*TR* = 2000 ms, *TE* = 28 ms, flip angle = 20°) sensitive to BOLD contrast. Each volume consisted of tilted axial slices (3 mm thick, 0.5 mm slice gap) that provided whole brain coverage. Anatomical T1-weighted images were acquired in the following space: *TR* = 2100 ms, *TE* = 10, slice thickness = 4 mm with no gap at a 90° flip angle. Head motion was limited using foam head padding.

FMRI block design analyses were carried out using multiple regression. Preprocessing was conducted using SPM5 (Wellcome Trust Center for Neuroimaging, www.fil.ion.ucl.ac.uk/spm). Echoplanar Images (EPIs) were realigned to the first volume of acquisition and then smoothed (8 mm 3D Gaussian kernel). Separate regressors were used to model each block-type. Each regressor was convolved with a canonical hemodynamic response function (HRF) used to model blood oxygen level dependent (BOLD) responses to trial blocks. A *t*-statistic was generated for each voxel, and a subsequent map (an SPM) was created. Linear contrasts were used to test the relative activation associated with conditions of interest. Resulting contrast maps reflected the differences in activation between the conditions at each voxel location. Significant voxels met both a whole brain threshold of *p* < 0.001, and a minimum Familywise Error-corrected cluster size threshold computed using the SPM data structure for each relevant contrast map (requiring minimum cluster sizes ranging from 60 to 100 contiguous voxels). Contrasts between normal chessboards minus random chessboards and for random chessboards minus normal chessboards were used for each of the subjects independently. We then performed a second-level analysis of the group activation for these contrasts. Finally, we performed a between groups analysis contrasting the experts and less experienced players on both the normal-minus-random contrast and random-minus-normal contrast in order to localize areas in which the effects of chessboard organization differed between groups.

## Results

### Behavioral results

Experts performed with significantly greater accuracy (*M* = 98%) than less skilled players (*M* = 84%) for normal chessboard stimuli, *t*_(19)_ = 3.09, *p* < 0.01. There were no group differences and high accuracy with random chessboards (experts: *M* = 89%, less skilled: *M* = 86%), faces (experts: *M* = 96%, less skilled: *M* = 93%), objects (experts: *M* = 95%, less skilled: *M* = 88%), and scenes (experts: *M* = 82%, less skilled: *M* = 86%).

The expert and less skilled groups varied on several questionnaire dimensions reported after the task, further establishing that the two groups differed in their processing of chessboard stimuli (evaluated with independent samples *t*-tests). Note that data were incomplete for some comparisons due to some participants not answering some questions. Looking first at the difficulty ratings, less skilled players reported significantly greater difficulty (*n* = 11, *M* = 4.27, 1–7 scale) with tracking normal chess stimuli than experts (*n* = 11, *M* = 2.05), *t*_(20)_ = 3.82, *p* < 0.001, but not with random chess stimuli (*M*'s = 4.18 and 4.52, respectively). There were no significant differences in reported difficulty for comparisons of experts and less skilled players on faces, scenes, or objects. We note that nine of the eleven experts reported that the random chessboards were more difficult to perceive than the normal chessboards, with the two remaining experts reporting that they were equally difficult. Only three of the eleven less skilled players reported that the random boards were more difficult to perceive than the normal boards with all others reporting equal difficulty.

With normal chessboards, experts reported tracking a greater number of pieces (*n* = 9, *M* = 21.22) than did less skilled participants (*n* = 11, *M* = 6.73), *t*_(18)_ = 4.75, *p* < 0.0001, as was expected given the difference in expertise. With random boards as well, experts reported tracking more pieces (*n* = 9, *M* = 10.28) than less skilled participants (*n* = 11, *M* = 5.00), though the difference was smaller and only marginally significant, *t*_(18)_ = 1.80, *p* = 0.08.

Many participants reported seeing groupings of pieces (i.e., chunks) within chessboards. Experts reported more pieces per grouping (*n* = 8, *M* = 9.69) than less skilled players (*n* = 10, *M* = 2.75), *t*_(16)_ = 2.15, *p* < 0.04. Looking at normal and random chessboards separately, there was also a marginally significant difference with experts reporting more groupings of pieces than did less skilled participants in normal chessboards (*n* = 8, 10, *M*'s = 2.94 and 1.45, respectively, *t*_(16)_ = 1.81, *p* = 0.08), but not in random chessboards (*M*'s = 1.80 and 1.00).

### Neuroimaging results

**Figure 2:**
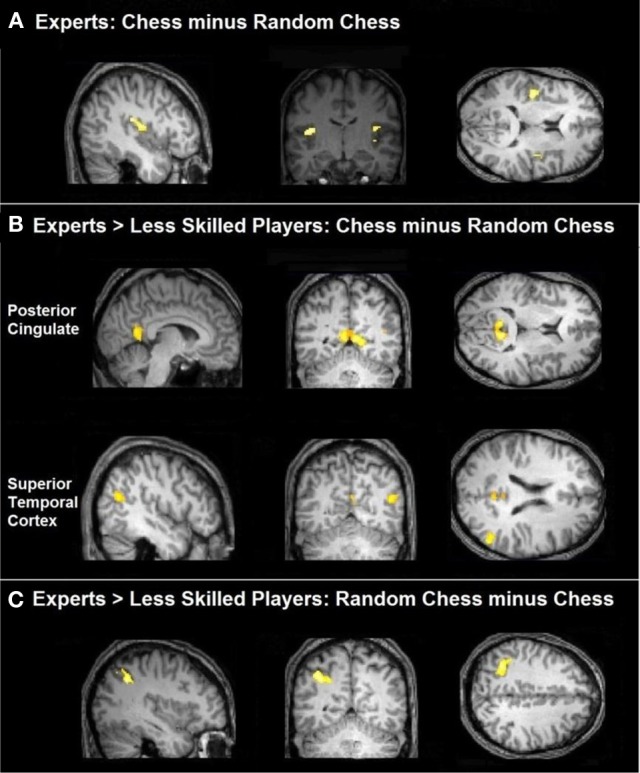
**Task-related activation comparing groups. (A)** Chess experts showed bilateral activation of the insula for the comparison of real chess perception minus randomly scrambled chess. **(B)** Chess experts differed from less skilled players in showing more activation in posterior cingulate and right superior temporal cortex when processing normal chess as compared to random chess. **(C)** Chess experts differed from less skilled players in showing more activation within the left parietal cortex when processing randomly scrambled chess as compared to normal chess.

**Figure 3:**
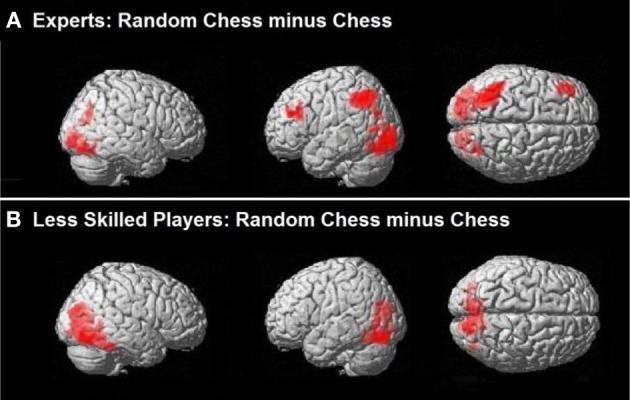
**Brain activation relevant to random chess. (A)** Regions of the bilateral occipital and parietal cortex were active for experts when contrasting randomly scrambled chess minus real chess. In addition the left DLPFC was active along with a left predominance in parietal activation. **(B)** Less skilled players showed bilateral occipital activation with active clusters extending into the inferior parietal cortex when contrasting randomly scrambled minus real chess.

Our initial comparisons were conducted independently on each group (experts and less skilled players). A normal—random contrast (i.e., the normal-minus-random-chess difference) showed activation in the bilateral insula among the experts (Figure 2A), but it did not show any significant regions of activation among the less skilled players. The reverse, random—normal, comparison resulted in extensive activation in the experts within the left inferior parietal lobe, the left middle frontal gyrus, the lingual gyrus of the occipital lobe bilaterally, the left cuneus, and the right temporal lobe (Figure 3A). By contrast, the less skilled players showed activation only within the bilateral fusiform gyrus for the random—normal comparison (Figure 3B, and refer to Table A1 in the Appendix for activation coordinates and cluster sizes). Note that the random—normal contrast showed extensive activation in the expert group but not the less skilled group within parietal and frontal cortical regions that overlap with regions of the prefrontal-parietal network identified by Bor and Owen (2007).

As a conservative test of the group differences, we compared the experts to the less skilled players with respect to both the normal—random difference (Figure 2B) and the random—normal difference (Figure 2C). The first of these comparisons identified two brain regions—the bilateral posterior cingulate and the right middle and superior temporal gyri—as those in which the normal—random difference was reliably greater in the expert group than in the less skilled group. The underlying pattern was that, with normal chessboards, experts exceeded less skilled players in mean percent signal change in both areas (*M*'s = 0.42 and 0.09, respectively, for the posterior-cingulate area, and −0.02 and −0.13, respectively, for the combined right temporal areas). These differences were absent or reversed with random chessboards (*M*'s = 0.10 and 0.11, respectively, for the posterior cingulate area and = −0.19 and −0.17 for the lateral temporal areas).

The second comparison identified a single brain region—the left inferior parietal cortex—as one in which random—normal difference was reliably greater in the expert group (Figure 2C). This area overlaps with the left inferior parietal region (the IPS) in Bor's frontal-parietal network. The next section examines the activation pattern in that region well as other frontal-parietal regions.

### Prefrontal-parietal region-of-interest results

**Figure 4:**
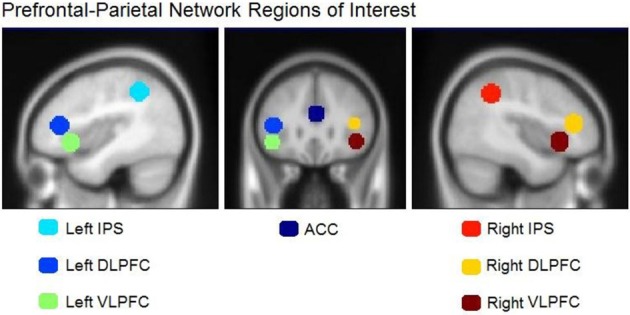
**Regions of interest in the prefrontal-parietal network as defined by Bor and Owen (2007)**. Included are four regions: **left** and **right** DLPFC, **left** and **right** VLPFC, **left** and **right** IPS, and the ACC.

The left inferior parietal region identified by our between-group analysis of the random—normal contrast is only one of seven areas that Bor and Owen (2007) linked to the prefrontal-parietal network. We sought to determine whether one or more of the remaining six areas might show a similar effect among experts—as compared to less skilled players—if examined with more sensitive ROI-based analyses. We created regions of interest (ROIs) using the coordinates and diameters specified by Bor and Owen (2007) based on prior reports from Duncan and Owen (2000), Bor et al. (2003, 2004), and Duncan (2006) using MarsBaR software (http://marsbar.sourceforge.net/). All ROIs were spherical with diameters of 10 mm and centers specified as follows in MNI coordinates: ACC (1, 33, 23), left DLPFC (−42, 33, 11), right DLPFC (39, 36, 13), left VLPFC (−43, 22, −6), right VLPFC (41, 22, −5), left IPS (−38, −47, 45), and right IPS (41, −47, 43). All ROI center coordinates were obtained by converting the Talairach coordinates reported in Bor and Owen (2007) to MNI using the Signed Differential Mapping software (http://www.sdmproject.com/utilities/?show=Coordinates). These regions are shown in Figure 4. ROI data were extracted and converted to percent-signal-change for each participant using MarsBar software (sourceforge.net/projects/marsbar, Brett et al., 2002). We extracted percent signal change for all ROIs for the chessboard and random chessboard conditions. We statistically evaluated the ROI data first by conducting 2 (group) × 2 (stimulus category) × 7 (area) ANOVA, which supported two interactions, an area by group interaction, *F*_(6, 120)_ = 3.09, *p* = 0.01, and an area × stimulus interaction, *F*_(6, 120)_ = 4.89, *p* = 0.0002. We followed this with separate group × stimulus ANOVAs for each of the seven areas.

The ANOVAs of the DLPFC and VLPFC ROIs yielded no significant effects. However, the VLPFC areas showed significant correlations that are described in the subsequent *correlational analyses* section.

**Figure 5:**
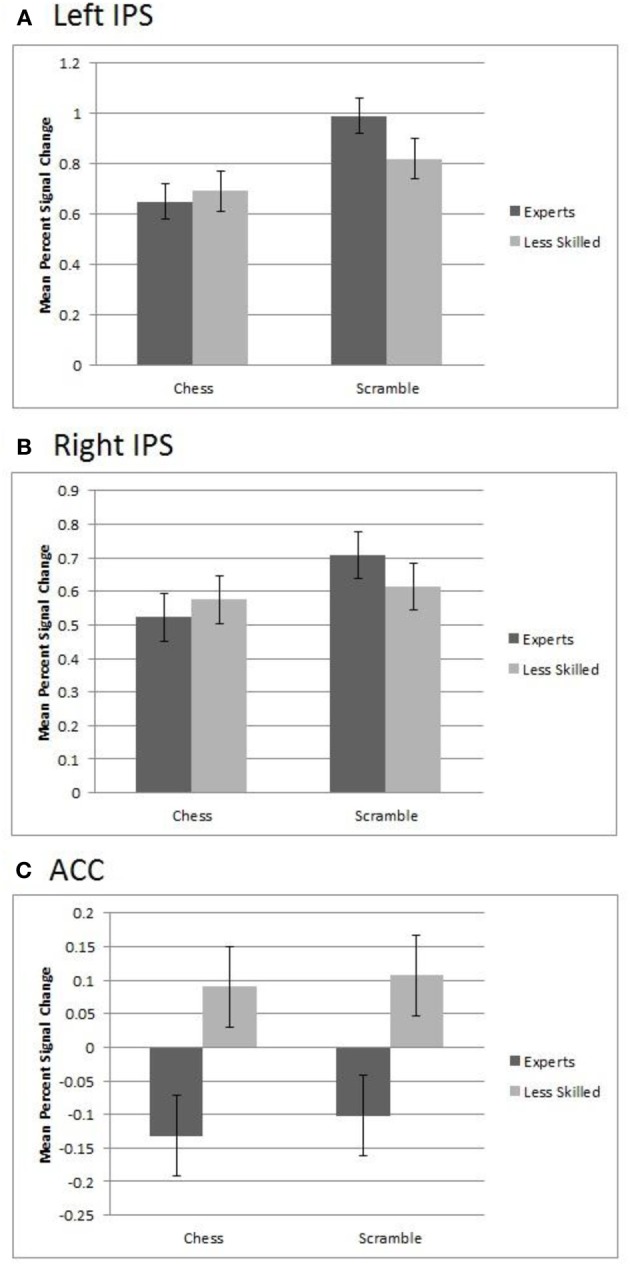
**Regions of interest in experts and less skilled players for randomly scrambled and normal chess. (A)** The left IPS showed greater activation for scrambled over normal chess and a group by category interaction. **(B)** The right IPS showed greater activation for scrambled chess over chess and a marginally significant interaction. **(C)** In the ACC, there was a significant main effect of group in which less skilled participants showed greater activation than experts.

In the ACC ROI, we observed a significant main effect of group, *F*_(1, 20)_ = 10.77, *p* = 0.004, in which less skilled participants showed greater activation (*M* percent signal change = 0.51) than experts (*M* = −0.12). This group effect was approximately equally strong with normal chess and random chess, as shown in Figure 5 (lower panel).

The IPS showed a different pattern, bilaterally differentiating the groups by stimulus category activation levels. The left IPS region showed a significant effect of category with random chessboards resulting in greater activation (*M* = 0.91) than normal boards (*M* = 0.67), *F*_(1, 20)_ = 31.32, *p* = 0.0001. It also showed a group by stimulus interaction, *F*_(1, 20)_ = 6.54, *p* = 0.02, such that the increased activation for random boards over normal boards was stronger among experts (*M*'s = 0.99 and 0.65, respectively), than among less skilled players, (*M*'s = 0.82 and 0.69 respectively), as shown in Figure 5 (upper panel). A similar but less robust pattern was observed in the right IPS, where there was a significant main effect of stimulus category, with random boards yielding greater average activation (*M* = 0.66) than normal chessboards (*M* = 0.55), *F*_(1, 20)_ = 9.06, *p* = 0.007, with a marginal group by stimulus interaction present, *F*_(1, 20)_ = 3.88, *p* = 0.06 (Figure 5, middle panel).

As the left IPS ROI overlaps with the region in which we previously found a reliable group difference in random-minus-normal activation levels (Figure 2C), the ROI-based analysis of this region confirms our prior observation that experts more than less skilled players show increased activation to random boards than normal boards. The analysis of the right IPS suggests that the group difference is largely if not completely bilateral. Finally, the ACC analysis shows that this interactive pattern obtained in the IPS regions does not extend to the entire prefrontal-parietal network, and that, moreover, at least one region within this network—the ACC—shows a strikingly different pattern.

The significant group x stimulus category interaction in left parietal cortex—and, by a less stringent criterion, right parietal cortex—suggests that parts of the prefrontal-parietal network may be relevant for the processing of random boards by experts. However, the theoretical significance of this pattern depends on whether experts' left parietal activation for random boards exceeds their left parietal activation not only for normal boards, but also for nonchess stimuli. We therefore, examined IPS activations in response to faces, scenes, and objects, in addition to random chessboards. The left IPS showed significant effects of both category, *F*_(3, 60)_ = 123.59, *p* < 0.001, and group (experts greater than novices), *F*_(1, 20)_ = 4.59, *p* < 0.05. Similar results were observed for the right IPS, with significant effects of category, *F*_(3, 60)_ = 63.43, *p* < 0.001, and group (experts greater than novices), *F*_(1, 20)_ = 69.02, *p* < 0.001. To establish whether the IPS activation to random boards was different than other categories in the experts, we conducted additional *post-hoc* comparisons (Bonferroni corrected *p* < 0.05). For the left IPS in experts, *post-hoc* comparisons revealed that random chess activation (*M* = 0.99) was higher than faces (*M* = 0.25), scenes (*M* = 0.29), and objects (*M* = 0.40). Similarly for the right IPS in experts *post-hoc* comparisons also showed that random chess activation (*M* = 0.71) was higher than faces (*M* = 0.13), scenes (*M* = 0.21), and objects (*M* = 0.20).

### Fusiform face area region-of-interest results

**Figure 6:**
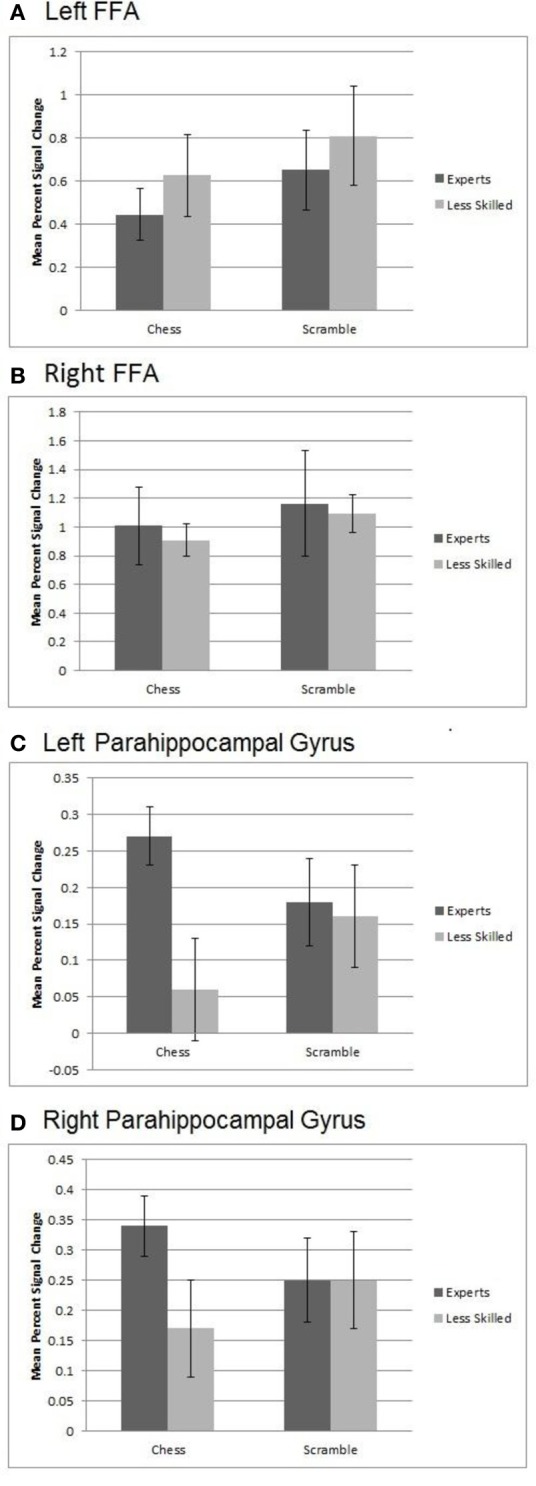
**Activation from Regions of Interest within the FFA and Parahippocampal gyrus. (A)** Left FFA activation showed a significant effect of stimulus category with the response to randomly scrambled chess being higher than that for real chess. **(B)** Similar results were observed for the right FFA with higher activation for random chess than real chess. **(C)** An interaction in the left parahippocampal gyrus with activation to normal chess for experts being higher than that for less experienced players. Both groups showed similar activation to randomly scrambled chess. **(D)** A marginally significant interaction in right parahippocampal gyrus with the activation to normal chess for experts being higher than that for less experienced players.

To evaluate effects of stimulus and group in the FFA, we created left and right FFA ROIs independently for each participant by defining maximally active voxels within the fusiform gyrus based on a contrast of face activation minus scene and object activation. Note that this ROI does not violate independence, as the defining contrast did not include either of the conditions to be evaluated in the ROI data (normal and random chess). We then conducted 2 (group) × 2 (normal chess, random chess) ANOVAs like those we performed on the prefrontal-parietal ROIs. As shown in Figure 6, the left FFA showed a significant effect of stimulus category, *F*_(1, 19)_ = 11.75, *p* < 0.01, with the FFA response to random chess (*M* = 0.73) being higher than that for normal chess (*M* = 0.54). Similar results were observed for the right FFA, *F*_(1, 19)_ = 6.49, *p* < 0.05, with higher activation for random chess (*M* = 1.13) than normal chess (*M* = 0.96). Neither ANOVA supported effects involving expertise.

### Medial-temporal region-of-interest results

To examine expertise and stimulus effects in medial temporal regions linked to long-term memory, we evaluated hippocampus and parahippocampal gyrus ROIs bilaterally. These ROIs were created by using the anatomically defined ROI from the WFU PickAtlas tool (Maldjian et al., 2003, 2004). A set of four ANOVAs supported no significant differences within the right or left hippocampus, but did support a significant interaction of stimulus category and expertise, *F*_(1, 20)_ = 6.75, *p* < 0.02, in the left parahippocampal ROI. As shown in Figure 6, left parahippocampal activation to normal chess for experts (*M* = 0.27) was higher than that for less experienced players (*M* = 0.06), while both groups exhibited very similar activation levels to random chess (experts *M* = 0.18, less experienced players *M* = 0.16). A similar, marginally significant, interaction emerged in right parahippocampal gyrus, *F*_(1, 20)_ = 3.96, *p* < 0.06, with the activation to normal chess for experts (*M* = 0.34) being higher than that for less experienced players (*M* = 0.17), and both groups similar for random chess (experts *M* = 0.25, less experienced players *M* = 0.25). These interactions are in line with prior evidence for activation increases in memory-related, medial-temporal regions with experts processing well-formed objects of expertise (Campitelli et al., 2007; Guida et al., 2012). Note also that the previously reported whole brain normal—random contrast (Figure 2B) supported similar patterns in the posterior cingulate and right middle and superior temporal brain regions.

### Correlational analyses

Of interest was whether activation differences between random chessboards and normal chessboards in the seven prefrontal-parietal ROIs might be correlated with (a) self-reported chunking-related activity, and (b) activation differences in the FFA and parahippocampal gyri. We computed a mean percent signal change difference score from each ROI for each participant by subtracting mean percent signal change from the normal chess condition from that of the random chess condition. We then computed inter-correlations (Pearson correlation coefficients, *r*) between these ROI difference scores for the five prefrontal-parietal areas and the following behavioral measures: mean number of groupings of pieces for random chess trials, mean number of pieces per grouping for random chess trials, and mean overall estimate of number of pieces tracked for random chess trials (the latter measure was strongly correlated with the product of the first two, *r* = 0.87). Total-pieces-tracked was reliably correlated with activation differences in both left IPS (*r* = 0.48, *p* < 0.05) and right IPS (*r* = 0.51, *p* < 0.05, *df* = 20 in all cases).

We next computed correlations between random—normal differences in the five prefrontal-parietal ROIs with random—normal differences in the FFA and parahippocampal gyri (*df* = 19 for correlations involving FFA, 20 for all others). Activation differences in the ACC were reliably correlated with activation differences in both left and right FFA and in both left and right parahippocampal gyrus (*r*'s = 0.54, 0.45, 0.46 and 0.52, respectively, all *p*'s < 0.05). Similarly, activation differences in the left VLPFC were correlated with activation differences the left FFA and left and right parahippocampal gyrus (*r*'s = 0.54, 0.59 and 0.52, respectively, *p*'s < 0.05). Finally, activation differences in the right VLPFC were correlated with activation differences in the left FFA (*r* = 0.49, *p* < 0.05). The correlations involving the ACC and VLPFC regions should not be viewed as independent, as activation differences in ACC were reliably correlated with activation differences in the left and right VLPFC (*r*'s = 0.60 and 0.48, respectively, *p* < 0.05), though activation differences in the left and right VLPFC were only weakly (and non-significantly) intercorrelated (*r* = 0.34). We note that activation differences in the left and right FFA and in the left and right parahippocampal gyri were strongly intercorrelated (*r*'s = 0.73 and 0.95, respectively, *p*'s < 0.01).

## Discussion

The main purpose of this study was to test the hypothesis that when experts encounter stimuli that fall within their skill-domain, but that are distorted in a manner which makes them impossible or in violation of rules in that domain, these experts engage an active search for structure akin to Bartlett's (1932) conception of effort after meaning. We framed this hypothesis in light of Bor and Seth's (2012) more recent theory linking conscious awareness to an active search for units or chunks, a process akin to effort after meaning. Bor's, 2012; theory links this active chunking process to neural activity in prefrontal and lateral parietal brain regions, and this theory is supported by recent fMRI studies showing that neural activation in these brain regions—known to be increased in a range of working memory and attentional tasks—are particularly increased in tasks involving active chunking of information.

Our focus here was on how extreme expertise might be related to this prefrontal-parietal chunking network in the domain of chess. Our starting point was the surprising observation that randomly scrambled chessboard displays, which violate the rules of chess and are known to disrupt expert performance, evoke as much if not more neural activation than do normal, meaningful displays in a ventral visual processing region linked to expertise, the FFA (Bilalić et al., 2011; Krawczyk et al., 2011). Considering other evidence that the prefrontal-parietal network exerts top-down control over ventral visual cortex activity (Tomita et al., 1999), including the FFA (Chadick and Gazzaley, 2011), we hypothesized that experts' processing of random displays, as compared to normal displays, should be linked to activation in prefrontal and lateral parietal brain regions, as well as in the FFA.

We tested this prediction with 11 master level chess experts and 11 less skilled players, all of whom performed a simple working memory task with normal chessboards, random chessboards, and three other types of stimuli using a blocked design. A whole-brain analysis identified a left IPS region that was reliably more active with random boards than normal boards in our expert group. This effect was not observed among our less skilled players, and, moreover, the random—normal difference in this region was significantly greater in the expert group than in the less skilled group (Figure 2). This observation was extended in a ROI analysis based on Bor and Owen's (2007) coordinates for seven subregions of the prefrontal-parietal network. In one of these regions, the left IPS, we found an interaction such that experts more than less skilled players showed increased activation for random boards over normal boards, confirming our finding from the whole-brain analysis (Figure 5, panel A). Activation in the right IPS showed a similar pattern, though it was less robust statistically (Figure 5, panel B).

The IPS data are in line with the hypothesis that expert chess players performing a working memory task respond to random boards by engaging an active search for novel chunks involving prefrontal-parietal network. In further support of this hypothesis, IPS activation among expert subjects not only was higher for random boards than normal boards; it also was higher for random boards than for three types of nonchess stimuli (faces, objects, and scenes). Finally, we observed correlations between random—normal activation differences in the left and right IPS and self-reports of the number of pieces tracked from random boards (a measure strongly correlated with product of the reported number of groups and the average size of groups). These two findings strengthen the case that IPS activation is functionally linked to an active search for chunks, in line with the active chunking hypothesis.

It may seem odd to propose that experts engaged in more chunking activity with randomly scrambled boards than normal boards, as the former are undoubtedly difficult for them to encode and might reasonably be expected to support the detection of fewer and/or smaller patterns or groups. Indeed, our post-test questionnaire data support this proposition. However, our proposal is more intuitive by a dual-mode view (Bor, 2012, pp. 152–156), a view that distinguishes the active search for and discovery of chunks from a more automatic process of identifying previously learned patterns. It is the former process that we suggest is supported by the IPS, and that is more strongly engaged in expert processing of random boards than normal boards. Of course, a change in the behavioral task from our simple working memory test to one more demanding of chess expertise might increase experts' active search for chunks in normal chess displays. The conditions in which experts engage an active search for structure have only begun to be explored.

Although an active chunking hypothesis is consistent our data from the IPS brain regions, it was not supported for five other components of the prefrontal-parietal network identified by Bor and Owen (2007). In none of these five regions did we find support (in the form of a group x stimulus category interaction) for experts showing increased activation for random boards than normal boards relative to less skilled players. In fact, in one of these regions—the ACC—we found a strikingly different pattern: In that region associated with detection of conflict and executive function (Botvinick et al., 1999, 2001; Botvinick, 2007), experts showed reliably less activation than less skilled persons with both normal and random boards (Figure 5, panel C). We did not predict this pattern, but it is consistent with much evidence for reduced activation in prefrontal brain regions as a function of expertise with the stimuli being processed (Hill and Schneider, 2006). The fact that our ACC data support this effect with random boards as well as normal boards might be viewed as contradicting this general expertise-deactivation relationship. However, Bilalić et al. (2010) have provided evidence that chess expertise entails object level processing (identifying individual chess pieces) as well as pattern-level processing (processing chess configurations). Thus, one possible resolution is that our ACC data reflect the effects of expertise on object level processing of chessboard pieces, which might proceed similarly with normal boards and random boards.

Another interesting difference between the ACC area and the IPS regions concerned correlations with the ventro-temporal FFA regions. We expected to observe correlations between random—normal differences in FFA activation and random—normal differences in prefrontal-parietal regions. Indeed, activation differences in the left FFA were reliably correlated with activation differences in the ACC, and in the left and right VLPFC areas. However, activation differences in the FFA were not reliably correlated with activation differences in the IPS areas. The pattern is puzzling, as the ACC region did not show an overall random—normal difference while the FFA and IPS regions did. Further, only the IPS regions supported the critical prediction of an increased random—normal difference in experts as compared to less skilled players.

There is much to learn. However, thinking more broadly, the striking difference between the IPS and ACC areas in the pattern of group and randomization effects underscores the importance of characterizing the functions of different prefrontal-parietal regions as they relate to expertise. It is possible that only some of these regions contribute to the active search for chunks, or that, alternatively, all contribute to active chunking, but in different ways. One plausible and readily testable hypothesis is that our simple, 1-back working memory task did not evoke a full-fledged active chunking strategy, but only one component of that strategy. There is evidence that the IPS region may be involved in the conjoint encoding or “binding” of features “intrinsic” to a stimulus (e.g., the color and spatial location of a word, Uncapher et al., 2006). In the case of chess displays, binding of color, spatial location and shape might constitute one component of an active chunking strategy, one that is engaged in simple, time-limited tasks, such as the 1-back task used here. In more complex and temporally extended tasks, other components of an active chunking strategy might become engaged, and expertise-related random—normal differences might emerge in other prefrontal-parietal brain regions.

A subsidiary goal of this study was to extend prior evidence bearing on the hypothesis that well-formed stimuli in a domain of expertise contain many familiar patterns that activate representations in long-term memory, allowing long-term memory to support performance in working memory tasks (Campitelli et al., 2007; Guida et al., 2012). An ROI analysis of the left and right parahippocampal regions supported this hypothesis: In both of these regions, known to be involved in recollection and memory for the spatial contexts of objects (Eichenbaum et al., 2007), chess expertise was linked to increased activation with normal boards, but not with random boards (see Figure 6). Our whole brain normal—random contrast supported a similar interactive pattern in the posterior cingulate and the middle and superior right temporal gyri (Figure 2B). Using a threat-detection task, Bilalić et al. (2012) observed a similar interactive pattern in both the parahippocampal and anterior cingulate regions, though in their case expertise effect was larger with normal than random boards, though it appeared to be present with both. More research is needed to better characterize the processes linked to these regions in expert chess processing, as there are several viable candidates including episodic memory retrieval, pattern recognition, visuospatial semantic memory retrieval, and self-referential/default network processing (discussions in Krawczyk et al., 2011 and Bilalić et al., 2012). For the present, the data from this and several other recent studies are consistent with the use of long-term memory in working memory tasks as one aspect of expertise. At the same time, the present study suggests that an active chunking strategy—or some component of that strategy—is engaged by experts when processing distorted structure in their domain of expertise.

### Conflict of interest statement

The authors declare that the research was conducted in the absence of any commercial or financial relationships that could be construed as a potential conflict of interest.

## Appendix

**Table A1:** **Independent whole-brain comparisons for Expert and Less Experienced player groups**.

**Anatomical region**	**Laterality**	**Voxels**	**Peak coordinates*x*, *y*, *z* (MNI)**
**EXPERTS**			
**Chess minus random chess**			
Insula	L	127	−40, −14, 14
			−42, −24, 20
Insula	R	91	40, −10, 2
			40, −20, 4
			44, −16, 18
**EXPERTS**			
**Random chess minus chess**			
Inferior parietal	L	1151	−30, −58, 42
			−26, −54, 36
			−40, −54, 46
Middle frontal gyrus	L	213	−44, 38, 26
			−34, 44, 34
			−42, 26, 22
Lingual gyrus (Occipital)	R	527	20, −82, −16
			24, −76, −12
			20, −94, −6
Cuneus (Occipital)	L	114	−28, −68, 6
			−16, −74, 10
Lingual gyrus (Occipital)	L	1439	−16, −82, −4
			−26, −76, −12
			−38, −84, −6
Temporal	R	118	32, −70, 26
			22, −76, 18
			34, −68, 36
**LESS EXPERIENCED PLAYERS**			
**Random chess minus chess**			
Fusiform gyrus	Bilateral	3595	−30, −78, −18
			32, −42, −24
			−38, −70, −14
**EXPERT > LESS EXPERIENCED PLAYERS**			
**Chess minus random chess**			
Posterior cingulate	Bilateral	22	10, −52, 8
		37	−8, −54, 10
			2, −60, 22
Middle temporal gyrus	R	14	46, −64, 20
		10	54, −66, 22
		40	46, −56, 18
**EXPERT > LESS EXPERIENCED PLAYERS**			
**Random chess minus chess**			
Inferior parietal lobule	L	291	−36, −56, 40
			−30, −62, 46
			−44, −46, 40
